# Identifying epigenetic aging moderators using the epigenetic pacemaker

**DOI:** 10.3389/fbinf.2023.1308680

**Published:** 2024-01-03

**Authors:** Colin Farrell, Chanyue Hu, Kalsuda Lapborisuth, Kyle Pu, Sagi Snir, Matteo Pellegrini

**Affiliations:** ^1^ Department of Molecular, Cell and Developmental Biology, University of California, Los Angeles, Los Angeles, CA, United States; ^2^ Department of Evolutionary Biology, University of Haifa, Haifa, Israel

**Keywords:** epigenetic, aging, epigenetic clock, DNA methylation, epigenome

## Abstract

Epigenetic clocks are DNA methylation-based chronological age prediction models that are commonly employed to study age-related biology. The difference between the predicted and observed age is often interpreted as a form of biological age acceleration, and many studies have measured the impact of environmental and disease-associated factors on epigenetic age. Most epigenetic clocks are fit using approaches that minimize the error between the predicted and observed chronological age, and as a result, they may not accurately model the impact of factors that moderate the relationship between the actual and epigenetic age. Here, we compare epigenetic clocks that are constructed using penalized regression methods to an evolutionary framework of epigenetic aging with the epigenetic pacemaker (EPM), which directly models DNA methylation as a function of a time-dependent epigenetic state. In simulations, we show that the value of the epigenetic state is impacted by factors such as age, sex, and cell-type composition. Next, in a dataset aggregated from previous studies, we show that the epigenetic state is also moderated by sex and the cell type. Finally, we demonstrate that the epigenetic state is also moderated by toxins in a study on polybrominated biphenyl exposure. Thus, we find that the pacemaker provides a robust framework for the study of factors that impact epigenetic age acceleration and that the effect of these factors may be obscured in traditional clocks based on linear regression models.

## 1 Introduction

Epigenetic clocks are accurate age prediction models based on DNA methylation that serve as promising tools for the study of aging and age-related biology. Beyond predicting the age of an individual to within a couple of years, multiple studies have shown that the difference between the observed and expected epigenetic age can be interpreted as a measure of biological age acceleration (Horvath and Raj, 2018). The first epigenetic clock was developed by Bocklandt et al. (2011). Since then, numerous epigenetic clocks have emerged. The pan-tissue Horvath clock (Horvath, 2013) and the blood Hannum clock (Hannum et al., 2013) are considered first-generation clocks. These first-generation clocks rely on a limited number of DNA methylation sites to estimate age and accurately predict an individual’s age across different tissues and cell types. GrimAge and DNAm PhenoAge are second-generation clocks, trained against biological age measures, enabling them to predict the mortality risk. As these age prediction models have gained popularity in human aging studies, they have been used to reveal health and environmental factors that impact the epigenetic age. These studies have led to the identification of multiple factors associated with a variety of health outcomes including mortality risk (Marioni et al., 2015; Perna et al., 2016), cancer risk (Dugué et al., 2018), cardiovascular disease (Huang et al., 2019), and other negative health outcomes (Horvath et al., 2014; Horvath et al., 2015; Armstrong et al., 2017). However, one intrinsic limitation underlying all of these epigenetic clocks is that as they predict age more accurately, epigenetic age acceleration effects become less significant (Zhang et al., 2019).

Epigenetic clocks are generally trained using a regularized regression model. Given an elastic net model of the form *y* = *βX*, the goal of penalized regression is to maximize the likelihood by reducing the prediction error of the model. However, sites where the relationship between methylation and time is non-linear may be discarded (Snir et al., 2019). Methylation sites that are associated with factors other than age (e.g., sex and cell type composition) that also increase the modeled error may also be discarded during model fitting. Therefore, these epigenetic models may not be optimal for detecting the effects of age moderating factors.

An alternative and complementary approach in studying epigenetic aging is to model how methylation for a predetermined collection of sites changes with respect to time. For this purpose, we have previously developed the epigenetic pacemaker (EPM) (Snir et al., 2016; Farrell et al., 2020) to model methylation changes with age. Under the EPM, the epigenetic state has a linear relationship with the modeled methylation data but not necessarily with chronological age. This allows for non-linear relationships between time and methylation to be modeled without prior knowledge of the underlying form.

In the current work, we ask whether the EPM formalism can be utilized for the identification of moderators that impact the association between age and the epigenetic state (i.e., factors that accelerate or decelerate the changes in epigenetic states with time). To this end, we extend the EPM model to simulate methylation matrices associated with age and age-accelerating phenotypes. We then evaluate the ability of regularized regression and EPM models to detect age acceleration traits that have linear and non-linear associations with age. Utilizing a large aggregate dataset, we validate the simulation results and, in one illustrative example, further assess the ability of the EPM to detect age-related methylation changes associated with PBB exposure.

## 2 Results

### 2.1 Simulation of trait-associated methylation matrices

To determine whether age-accelerating factors can be detected in synthetic data, we developed a simulation framework that allowed us to explicitly model epigenetic age-accelerating factors. In our simulation, we first define the age-associated phenotypes and then we derive the methylation levels that are consistent with these phenotypes. Simulated traits included a binary phenotype (*γ* = 0.5) and continuous phenotypes influenced by only age, or by age and sample factors (Table 1). We chose these trait forms as the binary phenotype simulates the effect of sex; the continuous phenotypes influenced by age only represent intrinsic epigenetic aging, and the continuous phenotypes influenced by sample-specific values represent individual characteristics, such as body mass index (BMI) or other disease-associated traits that could potentially impact epigenetic aging. The effect, *q*, of the binary trait was varied from 0.995 to 1.0 over five equally spaced intervals. For the non-binary traits with a non-linear age association, we used the form

$$p_{k,j}=Age_{j}^{0.5}q_{k,j}.$$ (1)

In this formula, a 0.001 decrease in *q* corresponds to a 1 percent decrease in the epigenetic state by age 100. Within each interval, the standard deviation of the sample parameter distribution was varied from 0.0 to 0.01 over five equally spaced intervals. The simulation was repeated 50 times for each combination of binary and continuous traits, with 500 simulated samples within each iteration. Additionally, at a binary *q*-value of 0.995, the range of continuous traits was expanded over a broader range to assess the model sensitivity for detecting the continuous trait. Five methylation sites for all continuous traits were then simulated and 50 methylation sites for the binary trait. Additional 50 sites were simulated that were equally influenced by a mixture of four continuous traits and the simulated binary trait. The resulting simulation matrix contains 450 methylation sites.

**TABLE 1:** Simulated trait conditions.

Trait form	Beta	Gamma	Gamma standard deviation	Sample effect	Age only	Generated phenotypes
Continuous	0.1	N(0.5,0.01)	0.05	Yes	No	10
Continuous	0.1	N(1.0,0.01)	0.05	Yes	No	10
Continuous	0.1	N(0.5,0.01)	0.05	No	Yes	20
Continuous	0.1	N(1.0,0.01)	0.05	No	Yes	20
Binary	0.1	0.5	0	Yes	No	1

Given a simulation dataset, the samples were split randomly in half for model training and testing. EPM models were fit for each simulation training set, and the epigenetic state and age predictions were made for the testing set. In the last step of our simulation, we asked whether we could identify whether the epigenetic state was impacted by the factors included in our model (i.e., whether we could detect age-accelerating and -decelerating factors). To determine the effect of each factor on the epigenetic state, we fit a regression model where the epigenetic age or state is dependent on the age, square root of the age, the continuous factor (e.g., BMI), and binary trait status of the sample.

$$S_{j}=Age+\sqrt{Age}+health_{j}+binary_{j}.$$ (2)

The square root of the age is included in the regression model to account for the non-linear relationship between the simulated age and methylation data.

**FIGURE 1:**
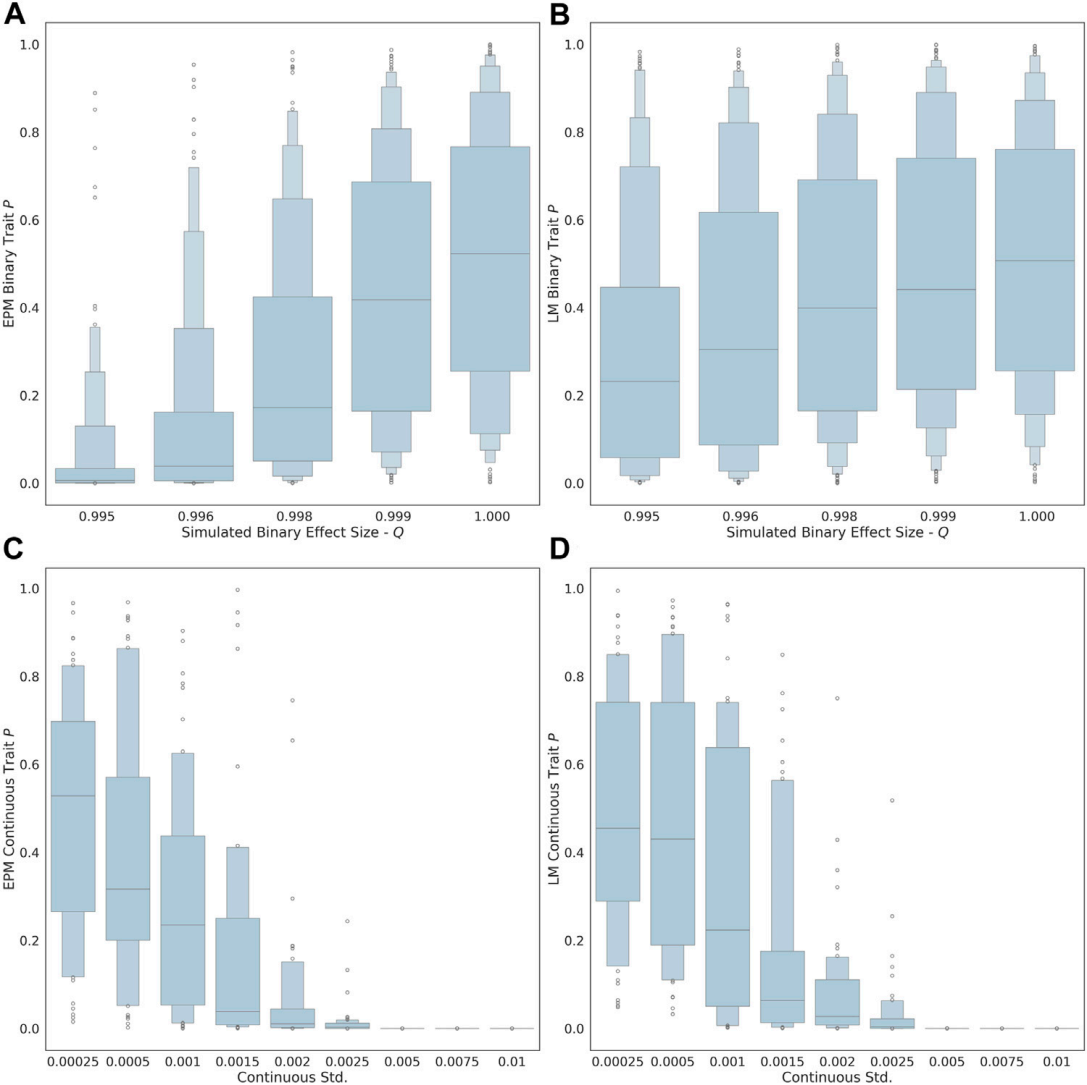
The distribution binary coefficient *p*-values for **(A)** EPM. **(B)** penalized regression models. The distribution of *p*-values given a simulation health standard deviation for **(C)** EPM and **(D)** penalized regression models.

As the exposure size (i.e., *q* value of each factor) of the binary trait is decreased from 1.00 to 0.995, the ability to detect the influence of the trait on the epigenetic state and age is improved (Figure 1A). At an effect size of 0.995, the estimated effect of the binary trait on the epigenetic state is significant (*μ* = 0.035, *σ* = 0.089). At an exposure size of 1.0, where the simulated binary trait has no effect, the distribution of *p*-values for EPM is not significant (i.e., *p* ≥ 0.05). The ability to observe the continuous factor effect of the simulated continuous traits improves in the EPM models as the standard deviation of the sample effect distribution is increased (Figure 1C). At an exposure size of 0.002 and 0.0025, the average EPM model is significant (*μ* = 0.0194, *σ* = 0.0436). At a continuous trait standard deviation above 0.005, the models produce significant results. This demonstrates that when the effect size is sufficiently large, we are able to identify epigenetic state accelerating and decelerating factors using our formalism.

We also explored whether we could identify moderators by computing the epigenetic age using more widely used linear models through penalized regression, as opposed to using the EPM. In this case, all the simulations steps were the same expect that instead of using the EPM to compute the epigenetic state, we used a penalized regression approach to estimate the epigenetic age of each individual. The main difference is that penalized regression leads to models where the epigenetic age is linear with the age. We found that the linear models are less sensitive for the detection of aging moderators than the EPM. At an effect size of 0.995, the estimated effect of the binary trait on the epigenetic state (i.e., EPM) is significant, while the effect on the epigenetic age (i.e., penalized regression) is not (*μ* = 0.269, *σ* = 0.282). Similarly, at an exposure size of 0.002 and 0.0025, the average EPM model is significant, while the average linear model is not (*μ* = 0.0607, *σ* = 0.128).

### 2.2 Universal blood epigenetic pacemaker and penalized regression models

We next repeated a similar analysis using a large aggregate dataset composed of Illumina 450K array data (Demetriou et al., 2013; Tan et al., 2014; Horvath and Ritz, 2015; Tserel et al., 2015; Voisin et al., 2015; Soriano-Tárraga et al., 2016; Dabin et al., 2020; Ventham et al., 2016; Marabita et al., 2018; Braun et al., 2019; Kurushima et al., 2019; Zannas et al., 2019; Johnson et al., 2020) deposited in the Gene Expression Omnibus (Barrett et al., 2012) (GEO), to determine whether we can identify aging moderators in real data. All methylation array datasets were processed using a unified pipeline from raw array intensity data (IDAT) files using minfi (Aryee et al., 2014). Sex and blood cell-type abundance predictions were made for each processed, as previously described (Houseman et al., 2012; Aryee et al., 2014). The aggregate dataset contains 6,251 whole-blood tissue samples, representing 16 GEO series.

We trained EPM and penalized regression models using data assembled from four GEO series (Johansson et al., 2013; Liu et al., 2013; Butcher et al., 2017; Dámaso et al., 2020) (*n* = 1605) with samples spanning a wide age range (0.01–94.0 years). The training set was split by predicted sex, and within each sex, we used stratified sampling by age to select 95% of the samples for model training. The selected samples from each sex were combined (*n* = 1524), and the remaining samples (*n* = 81) were left out for model evaluation. Methylation values for all samples were quantile-normalized by the probe type (Horvath, 2013) using the median site methylation values across all training samples for each methylation site. The cell-type abundance estimate usually leads to the prediction of about half a dozen cell types. In order to reduce the parameters in our moderation analysis, we used principal component analysis (PCA) to describe the cell types using only three components. The trained PCA model was used to predict the cell-type PCs for the testing and validation datasets.

**FIGURE 2:**
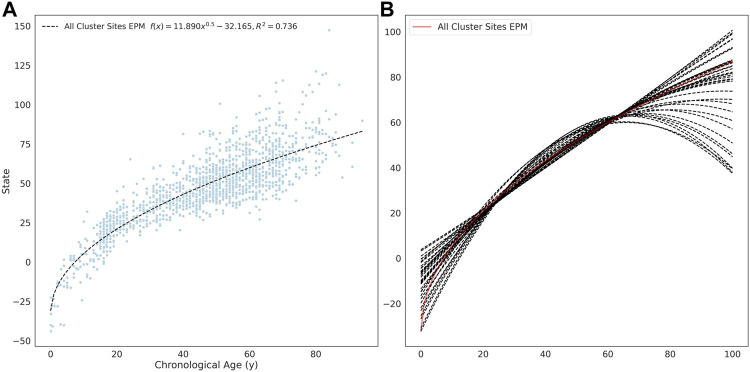
**(A)** EPM model fit with 3832 methylation sites with a MAE below 0.025. **(B)** The fit trend line for EPM clusters with more than 10 sites and an *R*_2_ ≥ 0.4.

The site selection for the EPM model is performed outside of model fitting. Methylation sites were selected for model training if the absolute Pearson correlation coefficient between methylation values and age was greater than 0.4 (*n* = 16, 880). A per site regression model was fit using the observed methylation value as the dependent variable and age as the explanatory variable. Sites with a mean absolute error (MAE) less than 0.025 between the predicted and observed methylation values were retained for further analysis (*n* = 7, 013). An EPM model was fit using these sites (Figure 2A). We then further filtered sites that lead to models with a low prediction error. To accomplish this, subsets of sites with a similar functional form were identified by clustering sites by affinity propagation (Frey and Dueck, 2007)) by the Euclidean distance between the single-site regression model residuals. Cross-validated EPM models were trained for all clusters with greater than 10 sites (*n* = 55). The cluster EPM models show varying associations between the epigenetic state and age relative to the EPM model fit with all sites initially selected by absolute PCC (Figure 2B).

In contrast to the EPM model, we fit the penalized regression model to the training matrix herein. The normalized training methylation matrix was first filtered to remove sites with a variance below 0.001, resulting in a training matrix with 183,114 sites. A cross-validated (*cv* = 5) elastic net model was trained against training sample ages using the filtered methylation matrix. The trained model performed well on the training (*R* ^2^ = 0.981) and testing (*R* ^2^ = 0.940) datasets (Supplementary Figures S1G,H).

Clusters with an observed EPM and penalized regression MAE less than 6 years (*n* = 5) were combined to fit final EPM and penalized regression models. This resembles the simulated methylation matrices where sites with differing functional forms are modeled collectively. The combined cluster EPM and the combined cluster regression model performed well on the training and testing datasets (Supplementary Figures S1A–C).

**FIGURE 3:**
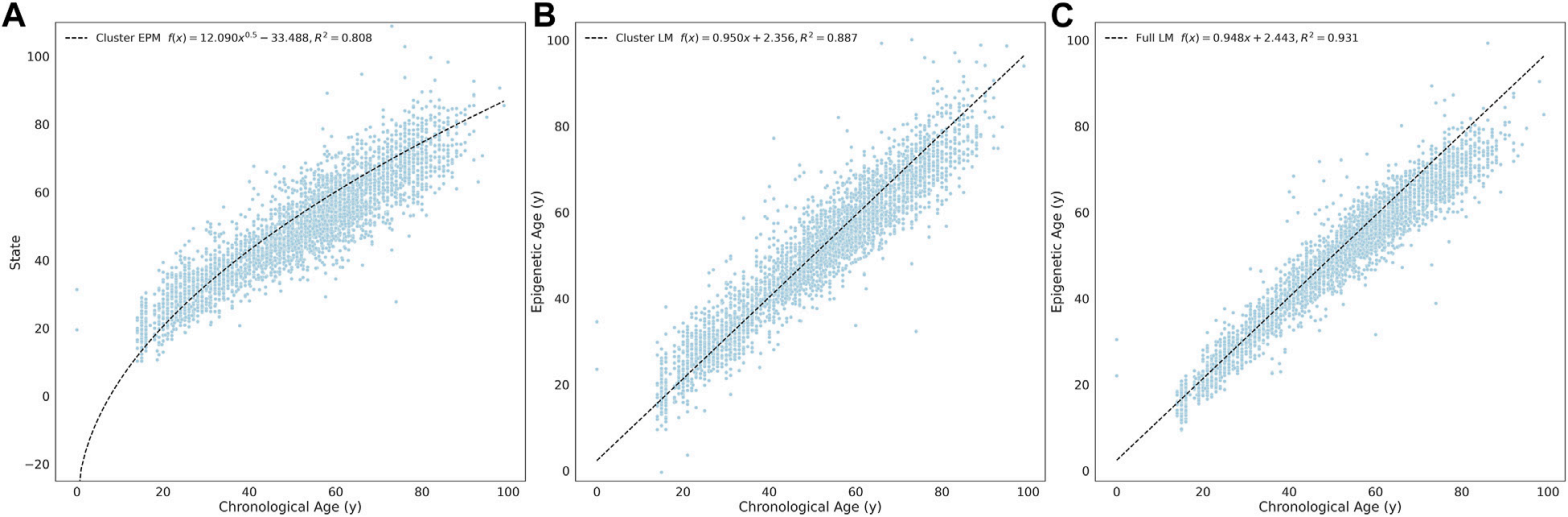
Whole blood tissue validation. **(A)** EPM. **(B)** cluster penalized regression and **(C)** full penalized regression models.

We evaluated the combined cluster EPM, combined cluster penalized regression, and the fully penalized regression models against a validation dataset consisting of 14 GEO series experiments, representing 4,600 whole-blood tissue samples. Each model accurately predicted the epigenetic state or epigenetic age of the validation samples (Figure 3).

In the last step, we attempted to identify moderators of epigenetic states (using the EPM) and epigenetic age (using penalized regression). To accomplish this, we fit an ordinary least squares regression model for every validation experiment individually to predict the observed epigenetic age or state using the sample age, the square root of age, cell-type PCs, and predicted sex:

$$S_{j}=Age+\sqrt{Age}+PC1+PC2+PC3+Sex+Intercept.$$ (3)

**FIGURE 4:**
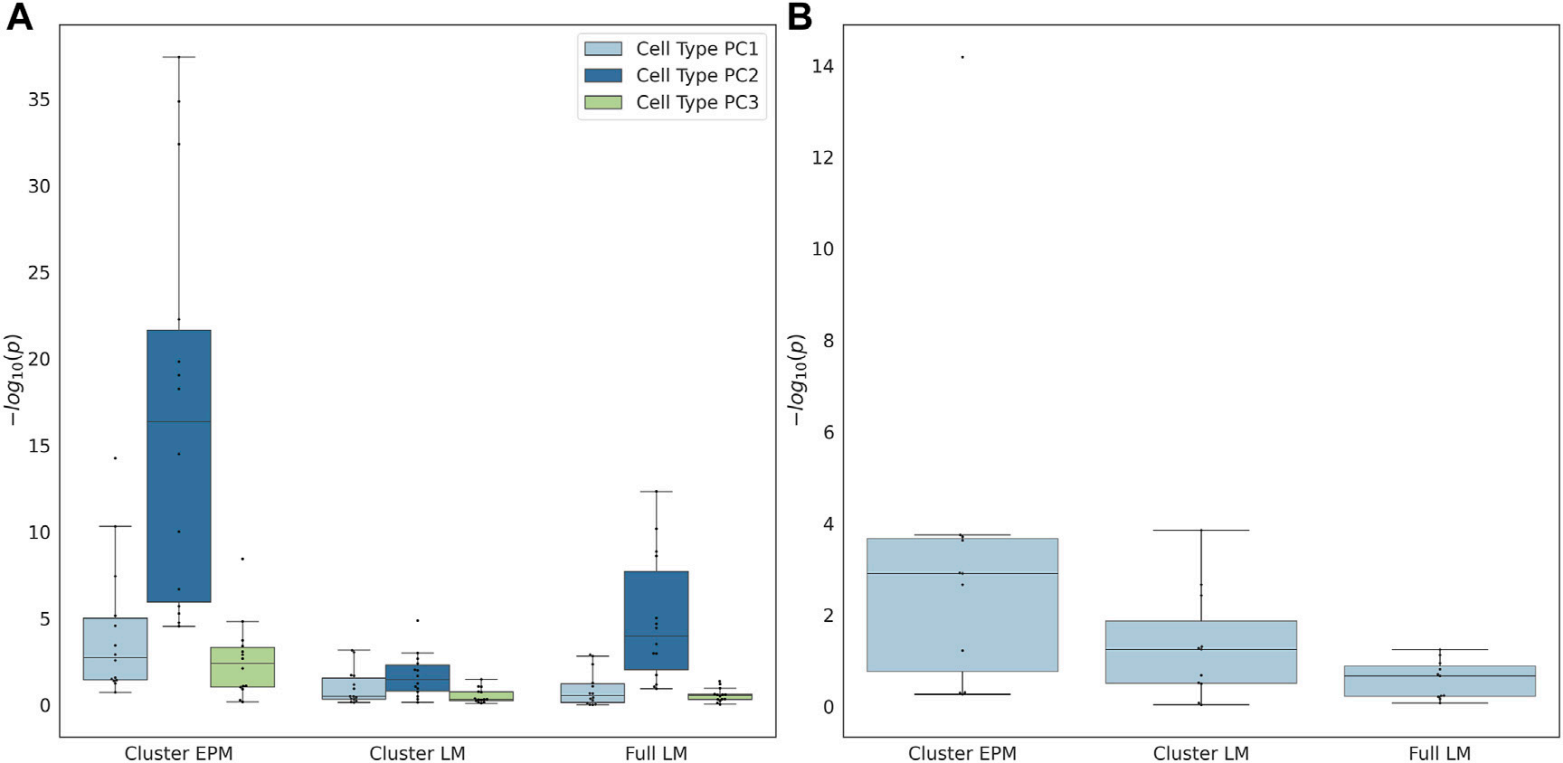
**(A)** Cell type principal component and **(B)** predicted sex regression coefficient *p*-values.

The individual terms were evaluated for significance to determine whether they significantly moderated the association between the epigenetic state or epigenetic age and the actual age. If the proportion of female samples to the total number of samples was greater than 0.7, the sex term was dropped from the regression model. The coefficients of the significant cell type PC2 were observed for all EPM models and the majority of the cluster and fully penalized regression models (Figure 4A). Significant cell-type PC1 and PC3 coefficients were observed for the majority of the EPM models but not for the cluster or fully penalized regression models. Significant sex effects (*p* < 0.0038) were observed for 9, 4, and 0 out of 15 models for the EPM, cluster penalized regression, and fully penalized regression, respectively (Figure 4B). This shows that, in general, the epigenetic state is more significantly impacted by sex and cell-type composition than the epigenetic age. Of course, we could not test for additional moderators in this dataset as we only computed the sex and cell-type composition of each sample. Therefore, we sought to identify additional datasets that included the measurement of factors that might impact epigenetic states or ages.

### 2.3 Polybrominated biphenyl exposure

Polybrominated biphenyls (PBBs) were widely used throughout the United States in the 1960s and 1970s for a variety of industrial applications. Widespread PBB exposure occurred in the state of Michigan from the summer of 1973 to later spring of 1974 when an industrial PBB mixture was incorrectly substituted for a nutritional supplement used in livestock feed (Fries and Kimbrough, 1985). PBB is biologically stable and has a slow biological half-life; individuals exposed during the initial 1973–1974 incident still have detectable PBB in their blood (Safe and Hutzinger, 1984). PBB is an endocrine-disrupting compound, and exposure has been linked to numerous adverse health outcomes in Michigan residents, such as thyroid dysfunction (Jacobson et al., 2017; Curtis S. W. et al., 2019) and various cancers (Hoque et al., 1998; Terrell et al., 2016). A study by Curtis et al. showed that the total PBB exposure is associated with altered DNA methylation at CpG sites, enriched for an association with endocrine-related autoimmune disease (Curtis S. W. et al., 2019). Utilizing the publicly available Illumina Methylation EPIC array (Pidsley et al., 2016) profiles (*n* = 679) that covered a wide age range (23–88 years), we sought to compare the ability of penalized regression and the EPM to detect epigenetic age acceleration associated with PBB exposure.

In brief, 50% of samples (*n* = 339) were selected for model training using stratified cross-validation by age. A cross-validated elastic net model was trained using all methylation sites with a site variance above 0.001, (*n* = 529, 703). The trained model performed well on the training and testing datasets (*R* ^2^ = 1.00, *R* ^2^ = 0.740, Supplementary Figures S2C,D). EPM sites were selected and models fit as described with the universal blood EPM. Four EPM clusters (*MAE* < 6) were merged for a combined EPM model built using 413 CpG sites. The combined EPM model performed well in training and testing datasets (*R* ^2^ = 0.794, *R* ^2^ = 0.812, Supplementary Figures S2A,B). Epigenetic age and epigenetic state predictions were then made for the testing samples using the penalized regression and EPM models. We then fit an OLS regression model

$$S_{j}=Age+\sqrt{Age}+PC1+PC2+PC3+Sex+PBB+Intercept$$ (4)

to predict the epigenetic age or state dependent on PBB exposure, age, the square root of age, cell-type PCs, and predicted sex. PBB exposure was highly significant in the EPM regression model (*p* = 5.9*e* − 10) but not the penalized regression model (*p* = 0.141).

## 3 Discussion

Epigenetic clocks are widely used biomarkers that can accurately predict the age of an individual based on their methylation pattern. They have been shown to be useful for human studies of aging and animal studies, including mice (Thompson et al., 2018) and dogs (Thompson et al., 2017). Epigenetic clocks are typically constructed using penalized regression approaches. Given a large enough matrix, penalized regression will select sites that minimize the prediction error. Beyond predicting actual ages, these models have also been used to measure the influence of external factors on the rates of aging, and multiple studies have shown that the resulting age accelerations (i.e., the differences between actual and predicted ages) are significantly associated with multiple factors such as cardiovascular disease (Huang et al., 2019) and mortality risk (Marioni et al., 2015; Perna et al., 2016).

Although epigenetic clocks have proven to be useful, they have significant limitations. Because they are based on linear models, it may be difficult to model aging when the underlying methylation changes are non-linear in time. Moreover, epigenetic clocks are prone to over-fitting, and while cross-validation schemes are often used to construct robust clocks, they often do not yield accurate estimates for some datasets. Finally, as epigenetic clocks become more accurate, they primarily predict age and will not be significantly affected by aging moderators. Therefore, more accurate epigenetic clocks become less useful in studying the impact of factors that accelerate epigenetic aging. This realization has led to the development of second-generation epigenetic clocks that are trained on health-adjusted aging measures rather than just ages (Levine et al., 2018; Lu et al., 2019).

To overcome some of these limitations, we have previously developed the EPM formalism. In this approach, rather than building a model for the age, we construct a model for the observed methylation data that depends on age. The advantage of this approach is that this formalism allows us to identify nonlinear associations between methylation and age across a lifespan. Moreover, these models tend to be robust to training as they are fit to large methylation matrices rather than age vectors. Finally, the model describes the change in methylation at each site with respect to a time-dependent epigenetic state, and therefore, all parameters of the model are directly interpretable as either initial values of methylation or rates of change of methylation.

Depending on the context, epigenetic clocks are both more and less effective than the EPM. Penalized regression models provide more accurate age predictions (*R* ^2^ = 0.875, 0.911) than the EPM model (*R* ^2^ = 0.821), and the model output can be directly compared to the age of a sample. However, because these models are optimized to reduce the error between the actual and predicted ages, they tend to minimize the effect of extraneous factors on the predicted age. As such, epigenetic clocks are not optimal for identifying external factors that moderate the relations between the actual and predicted ages. By contrast, the EPM models are not optimized to minimize the difference between the predicted and actual ages but rather try to minimize the difference between observed and modeled methylation values. As such, they retain many of the effects that other factors may have on the association between methylation and epigenetic states.

In this study, we find that while the penalized regression models were more accurate for predicting age, the epigenetic state generated by the EPM is significantly impacted by cell type and sex effects in both simulations and real data. Depending on the goal, an epigenetic measure that is sensitive to the cell type may or may not be advantageous. However, if one is interested in a cell-type independent measure of epigenetic age, the predictions can always be corrected using the inferred cell types. It is generally of greater interest to identify non-cell-type factors that influence the epigenetic age. To this end and as an example, we found that the EPM model generated for individuals exposed to PBB was sensitive to PBB exposure, which has been linked to negative health outcomes, while the penalized regression epigenetic aging model was not. Additionally, the sensitivity of the EPM to moderators of epigenetic aging has been supported by two recent studies investigating epigenetic aging in marmots (Pinho et al., 2021) and zebras (Larison et al., 2021). In the first of these studies, the EPM models showed an association between hibernation and slowed epigenetic aging in marmots and in the second an increased epigenetic age associated with zebra inbreeding; no such associations were observed with penalized regression epigenetic age models.

Most studies of human epigenetic aging are not motivated by the development of accurate age predictors since ages are nearly always known in studies but rather by the discovery of biological aging moderators. We, therefore, suggest that the EPM may be a more sensitive approach than epigenetic clocks for the detection of factors other than age that influence the epigenome and, therefore, potentially more useful for discovering moderators of biological aging.

## 4 Methods

### 4.1 Elastic-net regression model

Previous epigenetic clocks have utilized elastic-net regression to build age prediction models in the form of

$$L\left(\lambda_{1},\lambda_{2},\beta \right)=|y-X\beta {|}^{2}+\lambda_{2}|\beta {|}^{2}+\lambda_{1}|\beta |.$$ (5)

In the case of epigenetic clocks, the likelihood is maximized by minimizing the difference between the observed and predicted age across subjects while optimizing the elastic-net penalties, *λ*_1_ and *λ*_2_, using cross-validation approaches. We implemented this regression using the elastic-net model found in the Python scikit-learn library.

### 4.2 Epigenetic pacemaker model

In our previously published method, Farrell et al. (2020), the EPM was developed to account for non-linear relationships between age and methylation. The EPM models’ individual methylation sites are expressed as

$${\hat{m}}_{ij}=m_{i}^{0}+r_{i}s_{j}+\epsilon_{ij},$$ (6)

where

${\hat{m}}_{ij}$ is the observed methylation value.

$m_{i}^{0}$ is the initial methylation value.

*r*_*i*_ is the rate of change.*s*_*j*_ is the epigenetic state.

*ϵ*_*ij*_ is a normally distributed error term.

*r*_*i*_ and $m_{i}^{0}$ are characteristic of the sites across all individuals, and the epigenetic state of an individual *s*_*j*_ is set using information from all modeled sites. Given an input matrix $\hat{M}=\left[{\hat{m}}_{i,j}\right]$, the EPM utilizes a fast conditional expectation maximization algorithm to find the optimal values of $m_{i}^{0}$ , *r*_*i*_, and *s*_*j*_ to minimize the error between the observed and predicted methylation values across a set of sites. This is accomplished by first fitting a linear model per site using age as the initial *s*_*j*_. *s*_*j*_ of the modeled samples is then updated to minimize the error between the observed and predicted methylation values. This process is performed iteratively until the reduction in error is below a specified threshold or the maximum number of iterations is reached.

### 4.3 Simulation

We began with the assumption that under the EPM, the epigenetic state for individuals *j* and *S*_*j*_ can be interpreted as a form of biological age that represents a weighted sum of aging-associated phenotypes:

$$S_{j}=\overset{n}{\underset{k=1}{\sum }}\alpha_{1}p_{1,j}+\cdots +\alpha_{k}p_{k,j}.$$ (7)

Under this model,

*α*_*k*_ is the weight of the phenotype *k*.

*p*_*k*,*j*_ is the value of the phenotype *k*.

Phenotypes here may contribute to increased or decreased aging, and when considered as a whole, they contribute to the overall aging rate observed for an individual.

As shown in Snir et al. (2019), the relationship between *p*_*k*,*j*_ and time is not necessarily linear. When simulating age-associated phenotypes, each phenotype can be represented as

$$p_{k,j}=Age_{j}^{\gamma_{k}}q_{k,j},$$ (8)

where

*γ*_*k*_ is a phenotype specific parameter shared among all individuals.

*q*_*k*,*j*_ represents the coefficient, or exposure, of the phenotype for an individual.

The observed phenotype is modeled as an interaction between age and an exposure of varying magnitude among individuals. If *γ*_*k*_ = 1, then the effect of phenotype is linear with age, while if 0 < *γ*_*k*_ < 1, then the effect is non-linear. In this formulation, we can also include non-age dependent traits by setting

*γ*_*k*_ = 0 and $p_{k,j}=q_{k,j}=Age_{j}^{0}q_{k,j}$ .

Furthermore, to assess the sensitivity of the EPM at detecting moderators of epigenetic aging (i.e., phenotypes that accelerate or decelerate the epigenetic state of an individual), we simulated a methylation matrix containing linear and non-linear age-associated traits of the form

$$p_{k,j}=Age_{j}^{N\left(0.5,0.01\right)}q_{k,j}$$ (9)

and

$$p_{k,j}=Age_{j}^{N\left(1,0.01\right)}q_{k,j}.$$ (10)

The trait *γ* parameter was generated by sampling from a normal distribution N(0.5,0.01) to generate traits with varying relationships with time (Supplementary Figure S3). Thus, in this simulation, we include both phenotypes that influence the epigenetic state in a non-linear age-associated manner and those that affect it in a linear fashion. Samples were simulated by assigning an age from a uniform distribution, U(0,100) . In this formalism, the *q*_*k*,*j*_ term is a sample specific factor that influences the magnitude and direction of the simulated age-accelerating trait.

We implemented the simulation framework as a Python package with NumPy (≥v1.16.3) (Harris et al., 2020) and scikit-learn (v0.24) (Pedregosa et al., 2011) as dependencies. A simulation run generates a trait-associated methylation matrix, and the samples are tied to the simulated traits. The simulation procedure is implemented as follows:

• Traits are initialized that contain the information about the trait relationship with age and a simulated sample phenotype. Given the structure $p_{k,j}=Age_{j}^{\gamma_{k}}q_{k,j}$ , *k* samples, and *j* traits, *γ* is the characteristic of the trait. When a sample is passed to a trait, a value of *q* is generated for the sample by sampling from a normal distribution with a variance characteristic of the simulation trait. Additionally, each trait can be optionally influenced by a characteristic measure of sample health, *h*_*j*_. Given a normally distributed trait $N\left(\mu ,\sigma^{2}\right)$ and a health effect *h*_*j*_, the sampled distribution for individual *j* is $N\left(\mu +h_{j},\sigma^{2}\right)$ . Continuous and binary traits can be simulated. If a binary trait is simulated, a *q*-value other than 1 is assigned at a specified probability.
• Samples are simulated by setting the age by sampling from a uniform distribution over a specified range and by setting a sample health metric *h* by sampling from a normal distribution centered on zero with a specified variance. Traits passed to a sample simulation object are then set according to the age and health of the sample. Simulated samples retain all the set phenotype information for downstream reference.
• Methylation sites are simulated by randomly setting the initial methylation value, maximum observable methylation value, the rate of change at the site, and the error observed at each site. Sites are then assigned traits that influence the methylation values at each site.
• Methylation values are simulated for each site for every individual, given the simulated phenotypes with a specified amount of random noise.

The simulation data were randomly split in half into training and testing sets. The EPM models were fit using the simulated methylation matrix against age. Penalized regression models were fit using scikit-learn (v0.24) (Pedregosa et al., 2011) ElasticNet (alpha = 1, L1_ratio = 0.75, and selection = random). All other parameters were set to their default values. Ordinary least squares regression, as implemented in statsmodels (0.11.1) (Seabold and Perktold, 2010), was utilized to describe the epigenetic state or age with the following form:

$$S_{j}=Age+\sqrt{Age}+health_{j}+{binary}_{j}.$$ (11)

The complete analysis is found in the EPMSimulation.ipynb supplementary file.

### 4.4 Methylation array processing

Metadata for Illumina methylation 450K BeadChip methylation array experiments deposited in the GEO database (Barrett et al., 2012) with more than 50 samples were parsed using a custom Python tool set. Experiments that were missing methylation BeadChip array intensity data (IDAT) files, made repeated measurements of the same samples, utilized cultured cells, or assayed cancerous tissues were excluded from further processing. IDAT files were processed using minfi (Aryee et al., 2014) (v1.34.0). Sample IDAT files were processed in batches according to GEO series and BeadChip identification. Methylation values within each batch were normal-exponential normalized using out-of-band probes (Triche et al., 2013). Blood cell-type counts were estimated using a regression calibration approach (Houseman et al., 2012), and sex predictions were made using the median intensity measurements of the X and Y chromosomes, as implemented in minfi (Aryee et al., 2014). Whole-blood array samples were used for downstream analysis if the sample median methylation probe intensity was greater than 10.5 and the difference between the observed and expected median unmethylated probe intensity is less than 0.4, where the expected median unmethylated signal is described by (*y* = 0.66*x* + 3.718).

### 4.5 Blood epigenetic pacemaker and penalized regression models

Methylation sites with an absolute Pearson correlation coefficient between methylation values and age greater than 0.40 and 0.45 for the unified whole blood and PBB datasets, respectively, were initially selected for EPM model training. A linear model was generated using NumPy polyfit (Harris et al., 2020) with age as the independent variable and methylation values as the dependent variable. MAE was calculated as the mean absolute difference between the observed and predicted meth values, according to the site linear models. A vector of residuals generated using these models were utilized for clustering by affinity propagation (Frey and Dueck, 2007), as implemented in scikit-learn (v0.24) (Pedregosa et al., 2011) with a random state of 1 and a cluster preference of −2.5. Cross-validated EPM and penalized regression models for the universal blood analysis were trained for all clusters containing greater than 10 sites. Clusters with an observed EPM and penalized regression MAE less than 6.0 were combined to fit the final EPM and regression models.

Penalized regression models were fit using scikit-learn (v0.24) (Pedregosa et al., 2011) ElasticNetCV (cv = 5 alpha = 1, l1_ratio = 0.75, and selection = random). All other parameters were set to their default values. PCA, as implemented in scikit-learn, was utilized with default parameters to perform PCA on training sample cell-type abundances. The trained PCA was utilized to calculate cell-type PCs for the testing and validation samples. Ordinary least squares regression, as implemented in statsmodels (0.11.1) (Seabold and Perktold, 2010), was utilized to describe the epigenetic state or age with the following form:

$$S_{j}=Age+\sqrt{Age}+CellTypePC1+CellTypePC2+CellTypePC3+Sex+Intercept.$$ (12)

The complete analysis is found in the EPMUniversalClock.ipynb supplementary file.

### 4.6 Analysis environment

Analysis was carried out in a Jupyter (Basu, 2023) analysis environment. Joblib (Varoquaux and Grisel, 2009), SciPy (Virtanen et al., 2020), Matplotlib (Hunter, 2007), Seaborn (Waskom, 2021), Pandas (McKinney, 2012), and tqdm (Da Costa-Luis, 2019) packages were utilized during analysis.

## Data Availability

Publicly available datasets were analyzed in this study. These data can be found here: the dataset links are described in the manuscript.
