# Supplementary figures and images for: Identifying epigenetic aging moderators using the epigenetic pacemaker
Source: Front Bioinform. 2024 Jan 3;3:1308680. doi: 10.3389/fbinf.2023.1308680 (PMC10791860; doi:10.3389/fbinf.2023.1308680)

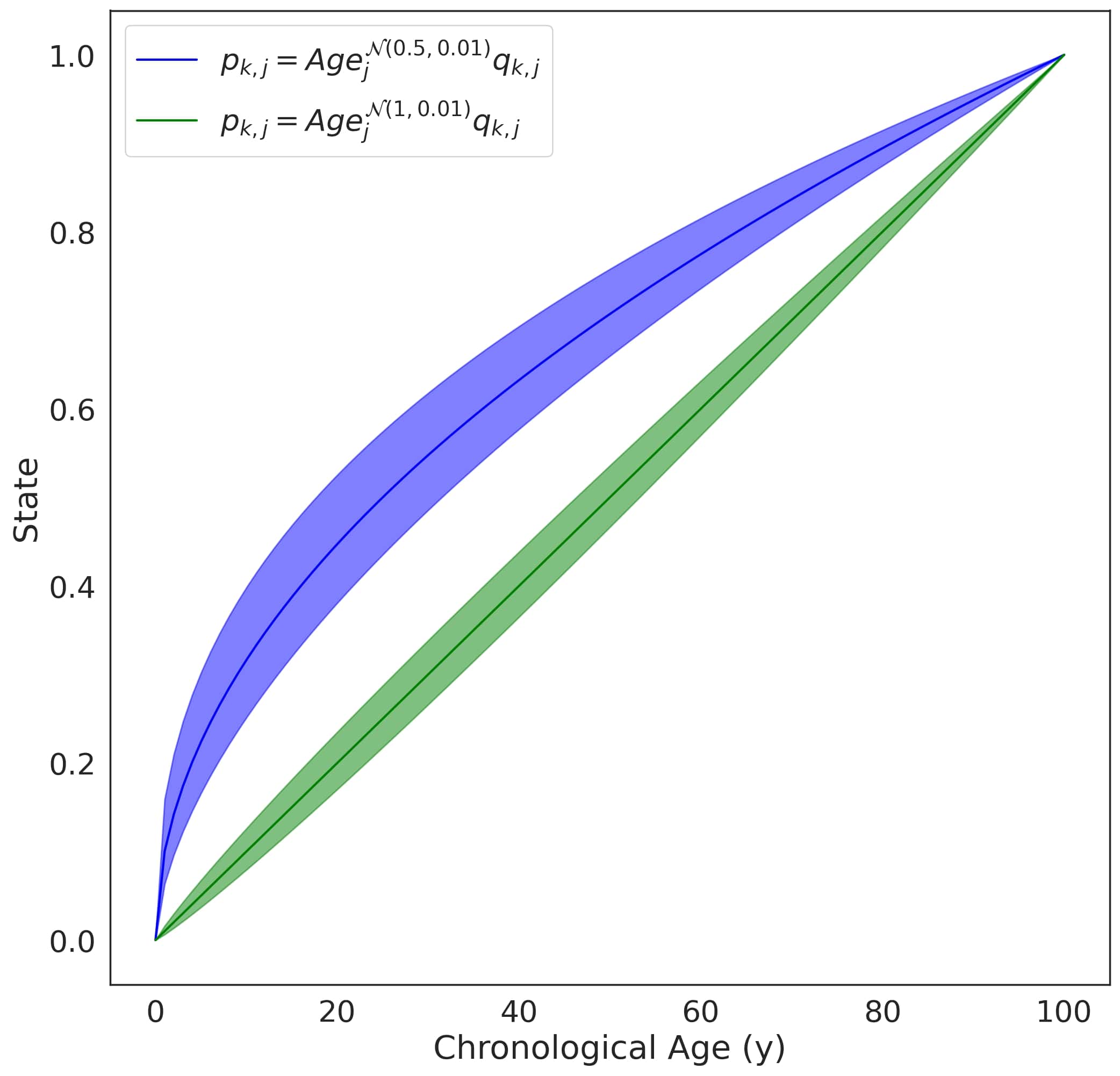

Supplement: Supplementary file 1 [file Image3.JPEG]

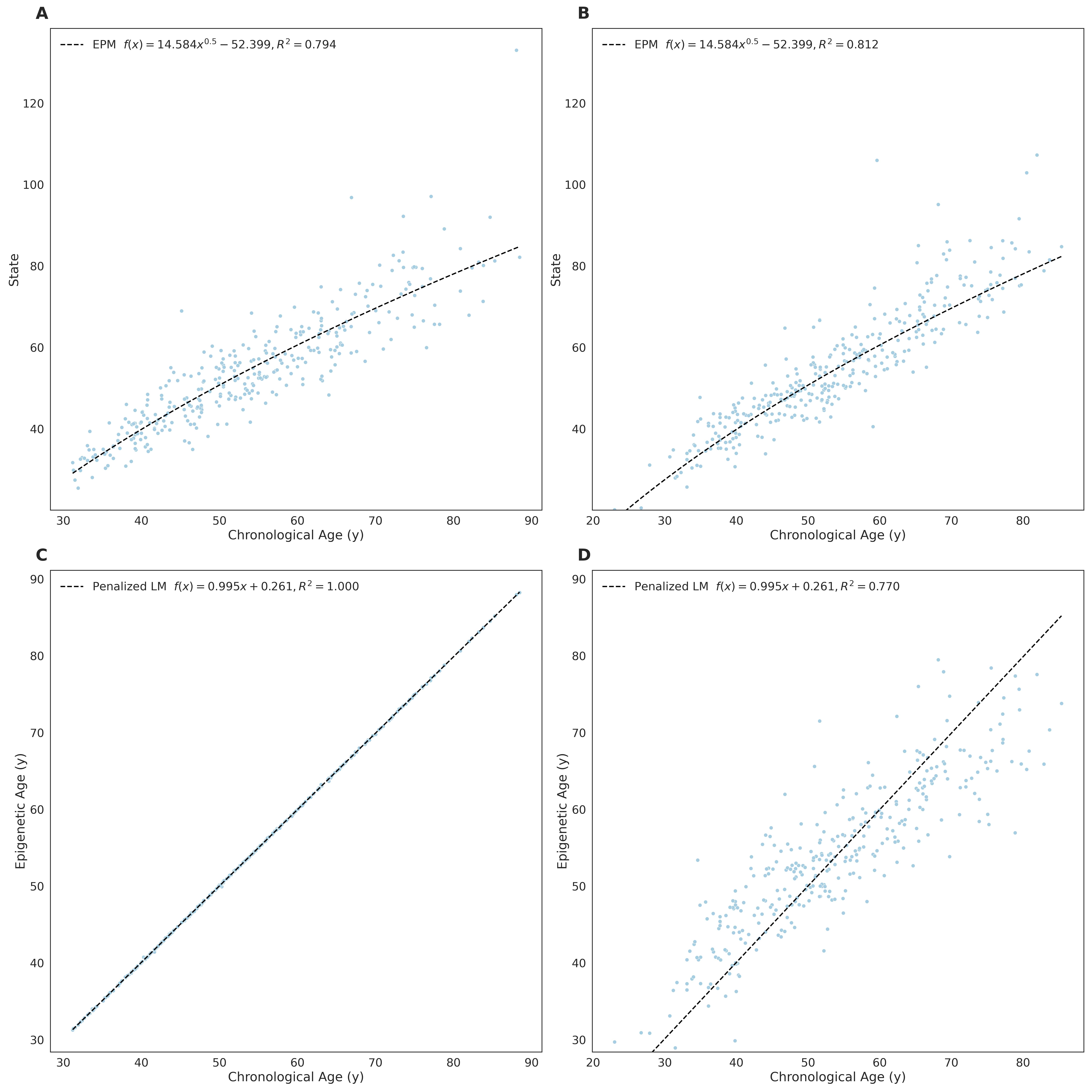

Supplement: Supplementary file 3 [file Image2.JPEG]
